# A Matron's Problem

**Published:** 1916-02-19

**Authors:** 


*To the Editor of* The Hospital.

### A Matron's Problem.

*To the Editor of* The Hospital.

Sir,—I should much like to know whether other matrons allow their sisters and nurses to smoke. The practice has now become so universal amongst educated women, and standards of professional conduct are changing so much in all directions, that I am wondering whether I am hopelessly old-fashioned and narrow-minded in refusing to allow my nursing staff to smoke, either in sitting or bed rooms, or, indeed, in any other place.—Believe me, yours faithfully, A Puzzled Matron.

February 14, 1916.

